# Independent Predictors of Mortality for *Aeromonas* Necrotizing Fasciitis of Limbs: An 18-year Retrospective Study

**DOI:** 10.1038/s41598-020-64741-7

**Published:** 2020-05-07

**Authors:** Tsung-Yu Huang, Kuo-Ti Peng, Wei-Hsiu Hsu, Chien-Hui Hung, Fang-Yi Chuang, Yao-Hung Tsai

**Affiliations:** 1 Division of Infectious Diseases, Department of Internal Medicine, Chang Gung Memorial Hospital, Chiayi, Taiwan; 2 Graduate Institute of Clinical Medical Sciences, College of Medicine, Chang Gung University, Taoyuan, Taiwan; 3 Department of Nursing, Chang Gung University of Science and Technology, Chiayi Campus, Chiayi, Taiwan; 4 Department of Orthopedic Surgery, Chang Gung Memorial Hospital, Chiayi, Taiwan; 5 Department of Chinese Medicine, School of Medicine, Chang Gung University, Chiayi, Taiwan; 6 Department of Laboratory Medicine, Chang Gung Memorial Hospital, Chiayi, Taiwan

**Keywords:** Diseases, Risk factors

## Abstract

Necrotizing fasciitis (NF) of the limbs caused by *Aeromonas* species is an extremely rare and life-threatening skin and soft tissue infection. The purpose of this study was to evaluate the specific characteristics and the independent predictors of mortality in patients with *Aeromonas* NF. Sixty-eight patients were retrospectively reviewed over an 18-year period. Differences in mortality, demographics data, comorbidities, symptoms and signs, laboratory findings, microbiological analysis, empiric antibiotics treatment and clinical outcomes were compared between the non-survival and the survival groups. Twenty patients died with the mortality rate of 29.4%. The non-survival group revealed significant differences in bacteremia, monomicrobial infection, cephalosporins resistance, initial ineffective empiric antibiotics usage, chronic kidney disease, chronic hepatic dysfunction, tachypnea, shock, hemorrhagic bullae, skin necrosis, leukopenia, band polymorphonuclear neutrophils >10%, anemia, and thrombocytopenia. The multivariate analysis identified four variables predicting mortality: bloodstream infection, shock, skin necrosis, and initial ineffective empirical antimicrobial usage against *Aeromonas*. NF caused by *Aeromonas* spp. revealed high mortality rates, even through aggressive surgical debridement and antibacterial therapies. Identifying those independent predictors, such as bacteremia, shock, progressive skin necrosis, monomicrobial infection, and application of the effective antimicrobial agents against *Aeromonas* under the supervision of infectious doctors, may improve clinical outcomes.

## Introduction

Necrotizing fasciitis (NF) is a rare and life-threatening necrotizing soft tissue infection (NSSTI) characterized by a rapid bacterial spread with soft tissue necrosis in the subcutaneous layers, particularly within superficial and deep fascia, with overall mortality rates of 12.1–76%^1–8^. Early fasciotomy, an appropriate empiric antimicrobial therapy supported by physicians specialized in infectious disease, and aggressive intensive unit care should be initially administered in critically ill patients suffering from fulminant NF to prevent limb loss and possible death^9–11^.

Our hospital is situated on the western coast of southern Taiwan, and most residents are exposed to occupations related to seawater, raw seafood, fresh or brackish water, and soil. As a result, *Vibrio* spp. and *Aeromonas* spp. infections have been reported at a relatively high incidence since 2004 in our hospital^9,12–19^. Thus, we set up the team “*Vibrio* NSSTIs Treatment and Research (VTR) Group”, which consists of professional staff working in various departments, including emergency medicine, orthopedic surgery, infectious diseases, intensive care unit (ICU), and hyperbaric oxygen treatment center. Our team has successfully decreased the mortality rate of *Vibrio* NF from 35% to 13%^8,12^.

Although we have established a treatment strategy including emergency fasciotomy or amputation, antibiotic therapy with a third-generation cephalosporin plus tetracycline, and admission to the ICU for patients suspected to have fulminant necrotizing fasciitis, such as *Vibrio*, MRSA, and *Aeromonas* infections^8,9,14,17,19^. *Aeromonas* species NSSTIs were still reported with a high mortality rate ranging from 26.7% to 100%^7–10,13,14,20–23^.

*Aeromonas* species are members of the *Vibrionaceae* and are gram-negative, non-sporulating, facultative, anaerobic small bacilli with a ubiquitous distribution^24,25^. Human infections including acute gastroenteritis, blood-borne infections, trauma-related skin and soft tissue infections (SSTIs), and intra-abdominal infections may develop in previously healthy subjects following aqueous environmental exposure^24–28^. Currently, there are more than 20 *Aeromonas* species identified, but only seven have been recognized as human pathogens, namely, *A. hydrophila*, *A. caviae*, *A. veronii biovar sobria*, *A. veronii biovar veronii*, *A. jandaei*, *A. trota*, and *A. schubertii*, with the first three being the most common causes of NF^24,29^ The aim of this study was to evaluate the specific characteristics and the independent predictors of mortality in patients with *Aeromonas* NF, and to gain insight and improve future outcomes.

## Results

### Patients outcomes

**Figure 1:**
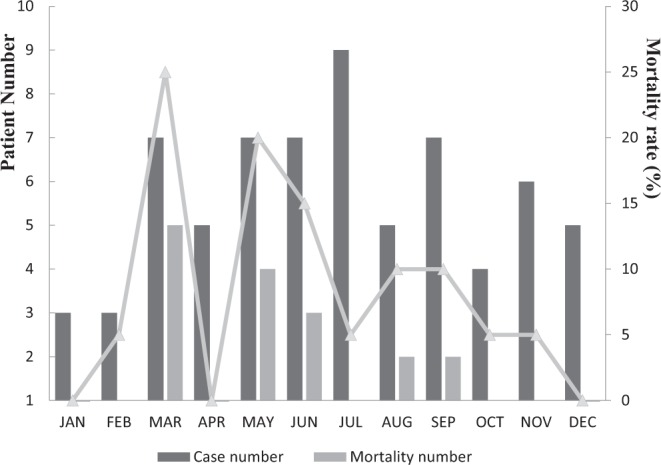
Monthly distribution of 68 cases of *Aeromonas* spp. necrotizing fasciitis of limbs in southern Taiwan.

From December 2001 to November 2019, a total 68 of patients admitted to the ED were surgically confirmed to have *Aeromonas* NF of limbs. Forty-eight patients survived, and 20 expired with a mortality rate of 29.4%. The numbers of diagnoses, cases of death, and mortality rates are listed per month (Fig. 1).

### Demographic data

No significant differences were observed within the parameters of age, gender, infective regions, or the number of amputations per patient between these two groups. The non-survival group was characterized by a significantly higher incidence of chronic kidney disease (CKD), chronic liver dysfunction, and ICU admission (Table 1). Meanwhile, the non-survival group had significantly associated with higher Acute Physiology and Chronic Health Evaluation II (APACHE^a^ II) scores, fewer operations, and shorter hospitalization stays (Table 1).

**Table 1:** Demographic data and characteristics from *Aeromonas* NF between survival and non-survival groups.

Variable	Survival (n = 48)	Non-survival (n = 20)	*p-*value
Age (years)	60.52 ± 15.44	64.85 ± 12.89	0.274
Gender, male	40 (83.3)	14 (70)	0.215
Involved region			
Upper extremities	12 (25)	1 (5)	0.056
Lower extremities	36 (75)	19 (95)	0.056
APACHE^a^ II score	12.48 ± 6.65	23.9 ± 7.33	<0.001*
Underlying chronic diseases			
Alcoholism	10 (20.8)	7 (35)	0.219
Chronic kidney disease	12 (25)	15 (75)	<0.001*
Cardiovascular disease	9 (18.8)	5 (25)	0.561
Cerebrovascular accident	5 (10.4)	2 (10)	0.959
Viral hepatitis	17 (35.4)	12 (60)	0.062
Liver cirrhosis	13 (27.1)	9 (45)	0.150
Chronic liver dysfunction	23 (47.9)	16 (80)	0.015*
Diabetes Mellitus	24 (50)	14 (70)	0.130
Malignancy	14 (29.2)	5 (25)	0.727
Peripheral vascular disease	7 (14.6)	4 (20)	0.580
Patients number of amputations	12 (25)	8 (40)	0.216
ICU^b^ admission	23 (47.9)	19 (95)	<0.001*
TiSO^c^ > 24 h	15 (31.3)	6 (30)	0.919
Number of debridement	3.5 ± 2.1	1.6 ± 1.0	<0.001*
ICU stay (day)	6.4 ± 14.7	13.5 ± 21.1	0.117
Hospital stay (day)	37.9 ± 20.2	17.1 ± 36.8	0.004*

Data were presented as mean (standard deviation) or frequency (%). *p-value <0.05. ^a^APACHE: Acute Physiology and Chronic Health Evaluation, ^b^ICU: intensive care unit, ^c^TiSO: time of the first surgical intervention from symptom onset to the operating room.

### Microbiological analysis

*Aeromonas hydrophila* was the most common infectious bacteria, accounting for 46 (67.6%) of the total 68 *Aeromonas* NF cases, followed by 10 *Aeromonas sobria* cases (14.7%), 9 *Aeromonas* cases (13.2%), and 3 of *Aeromonas caviae* (4.4%). A total of 42 (61.8%) of these patients were diagnosed with polymicrobial *Aeromonas* NFs of limbs. The most common isolates obtained from patients with polymicrobial infections included *Clostridium* species (21, 50.0%), followed by *Enterobacter* species (14, 33.3%) and *Klebsiella* species (11, 26.2%) (Table 2). The non-survival group had a higher incidence of bacteremia (70% vs. 25%; *p* = 0.001) than the survival group, and were significantly associated with monomicrobial infection (*p* = 0.018). Meanwhile, the survival group had a higher incidence of polymicrobial infection and coinfection with anaerobic pathogens (*p* = 0.017 and *p* = 0.016, respectively) than the non-survival group. Concerning antibiotic resistance to *Aeromonas* species, only resistant to cephalosporins exhibited a statistically significant increase (40% vs. 14.6%; *p* = 0.021) within the non-survival group. In terms of initial ineffective empirical antimicrobial usage, the non-survival group had a higher incidence (45% vs. 18.8%; *p* = 0.025) than the survival group (Table 3).

**Table 2:** Summary of identified infectious microorganisms in 42 cases of *Aeromonas* polymicrobial NF of limbs.

Identified infectious microorganisms	Total No. (%)
Gram-negative aerobic pathogens	
*Enterobacter* spp.	14 (33.3)
*Enterobacter cloacae*	13 (31.0)
*Enterobacter aerogenes*	1 (2.4)
*Klebsiella* spp.	11 (26.2)
*Klebsiella pneumoniae*	7 (16.7)
*Klebsiella oxytoca*	4 (9.5)
*Pseudomonas aeruginosa*	10 (23.8)
*Escherichia coli*	9 (21.4)
*Proteus vulgaris*	4 (9.5)
*Citrobacter freundii*	3 (7.1)
*Serratia marcescens*	2 (4.8)
*Vibrio vulnificus*	1 (2.4)
*Morganella morganii*	1 (2.4)
*Acinetobacter baumannii*	1 (2.4)
Gram-positive aerobic pathogens	
*Enterococcus* spp.	9 (21.4)
*Enterococcus faecalis*	8 (19.0)
*Enterococcus casseliflavus*	1 (2.4)
*Staphylococcus* spp.	4 (9.5)
MRSA^a^	2 (4.8)
MSSA^b^	2 (4.8)
Group D *Streptococcus*	1 (2.4)
*Anaerobic pathogens*	
*Clostridium* spp.	21 (50.0)
*Clostridium bifermentans*	9 (21.4)
*Clostridium* sp	6 (14.3)
*Clostridium perfringens*	3 (7.1)
*Clostridium bifermentans*	1 (2.4)
*Clostridium butyricum*	1 (2.4)
*Clostridium novyi A*	1 (2.4)
*Peptostreptococcus* spp.	7 (16.7)
*Peptostreptococcus anaerobius*	4 (9.5)
*Peptostreptococcus* sp	2 (4.8)
*Peptostreptococcus magnus*	1 (2.4)
*Bacteroides fragilis*	3 (7.1)
*Prevotella* sp	3 (7.1)
Total	42 (100)

Abbreviations: ^a^MRSA: methicillin-resistant Staphylococcus aureus.

^b^MSSA: methicillin-sensitive *Staphylococcus aureus*.

**Table 3:** Microbiological results for *Aeromonas* species NF between survival and non-survival groups.

Variable	Survival (n = 48)	Non-survival (n = 20)	*p-*value
Bloodstream infection	12 (25)	14 (70)	0.001*
Bacteria isolated			0.018*
Polymicrobial infection	34 (70.8)	8 (40)	
Monomicrobial infection	14 (29.2)	12 (60)	
Coinfection with anaerobic pathogens	22 (45.8)	3 (15)	0.016*
Coinfection with *Clostridial* spp.	17 (35.4)	2 (10)	0.033*
Antibiotics that *Aeromonas* spp. resistant			
Penicillins	19 (39.6)	5 (25)	0.252
Carbapenems	16 (33.3)	7 (35)	0.895
Cephalosporins	7 (14.6)	8 (40)	0.021*
Aminoglycosides	6 (12.5)	5 (25)	0.202
Sulfa drugs	4 (8.3)	5 (25)	0.065
Tetracycline	3 (6.3)	4 (20)	0.089
Fluoroquinolones	1 (2.1)	1 (5)	0.517
Other class antibiotics	36 (75)	17 (85)	0.365
Initial ineffective empirical antimicrobial usage	9 (18.8)	9 (45)	0.025*

### Clinical presentations

No significant differences in the presentation of fever (>38 °C); tachycardia (heartbeat >100/min); or erythematous, swollen, and painful lesion were observed between the two groups (Table 4). However, the proportion of patients in the non-survival versus survival group presenting with tachypnea (respiratory rate >20/min, 70.0% vs. 37.5%; *p* = 0.014), shock (systolic blood pressure <90 mmHg, 80.0% vs. 29.2%; *p* < 0.001), hemorrhagic bullae (60.0% vs. 29.2%, *p* = 0.017), and skin necrosis (55.0% vs. 16.7%, *p* = 0.001) were higher (Table 4).

**Table 4:** Comparison of clinical presentations between survival and non-survival groups.

Variable	Survival (n = 48)	Non-survival (n = 20)		*p-*value
Systemic symptoms/signs				
Fever (>38 °C)	15 (31.3)	5 (25)	0.606	
Tachycardia^a^	27 (56.3)	15 (75)	0.147	
Tachypnea^b^	18 (37.5)	14 (70)	0.014*	
Shock^c^	14 (29.2)	16 (80)	<0.001*	
Limbs symptoms/signs				
Pain and tenderness	47 (97.9)	20 (100)	0.516	
Swelling and erythema	45 (93.8)	20 (100)	0.253	
Hemorrhagic bullae	14 (29.2)	12 (60)	0.017*	
Skin necrosis	8 (16.7)	11 (55)	0.001*	

Data were presented as mean (standard deviation) or frequency (%). ^a^Tachycardia: heart beat >100/min, ^b^Tachypnea: respiratory rate >20/min, ^c^Shock: systolic blood pressure <90 mmHg.

### Laboratory findings

Within the non-survival group the following characteristics were observed more frequently than within the survival group: total white blood cell count <4000/uL, band leukocyte cells >10%, lymphocyte count of leukocytes <1000/uL, anemia (hemoglobin <10 g/dL), thrombocytopenia (platelet count <15 × 10^4^/uL), and estimated glomerular filtration rate <30 c.c./min (Table 5). In addition, the proportion of patients presenting with a lower albumin level was frequently observed and significantly higher in the non-survival group (*p* = 0.002). The prothrombin time (PT) and total bilirubin values for the non-survival group were significantly higher than those for the survival group (Table 5).

**Table 5:** Laboratory findings of patients with *Aeromonas* NF between survival and non-survival groups.

Variable	Survival (n = 48)	Non-survival (n = 20)	*p-*value
Total WBC^a^ count			
Leukocytosis (≧12000/uL)	28 (58.3)	6 (30)	0.033*
Leukopenia (≦4000/uL)	2 (4.2)	6 (30)	0.003*
Leukocytosis or Leukopenia	30 (62.5)	12 (60)	0.847
Differential count			
Neutrophilia (>7500/uL)	38 (79.2)	9 (45)	0.005*
Band forms (>10%)	7 (14.6)	8 (40)	0.021*
Lymphocytopenia (<1000/uL)	21 (43.8)	15 (75)	0.019*
Anemia (Hb^b^ <10 g/dL)	11 (22.9)	12 (60)	0.003*
Thrombocytopenia (platelet counts <15 × 10^4^/uL)	20 (41.7)	16 (80)	0.004*
eGFR^c^ < 30 c.c./min	9 (18.8)	14 (70)	<0.001*
Glucose (mg/dL)	191.0 ± 116.6	190.3 ± 116.2	0.983
Sodium (meq/L)	137.4 ± 4.4	135.1 ± 5.9	0.077
C-reactive protein (mg/dL)	111.9 ± 103.8	146.0 ± 163.6	0.336
Albumin (mg/dL)	2.8 ± 0.9	2.0 ± 0.6	0.002*
PT^d^ (s)	13.1 ± 3.3	19.5 ± 7.5	<0.001*
Total bilirubin (mg/dL)	2.1 ± 2.0	6.2 ± 5.4	<0.001*

Data were presented as mean (standard deviation) or frequency (%). Abbreviations: ^a^WBC: white blood cell; ^b^Hb: hemoglobin; ^c^eGFR: estimated glomerular filtration rate; ^d^PT: prothrombin time.

### Multivariate analysis

As determined by multivariate analysis, patients presented with bloodstream infection (OR: 8.741; 95% CI: 1.936–39.476; *p* = 0.005), shock(OR: 5.926; 95% CI: 1.254–28.006; *p* = 0.025), skin necrosis (OR: 4.575; 95% CI: 1.190–17.597; *p* = 0.027), and a higher incidence of initial ineffective empirical antimicrobial usage (OR: 5.798; 95% CI: 1.247–26.951; *p* = 0.025) were the indicators of mortality (Table 6).

**Table 6:** Multivariate regression for the non-survival group from 68 cases of *Aeromonas* NF patients about microbiological results and clinical presentations.

	OR^a^ (95% CI^b^)	*p-*value
Microbiological results		
Bloodstream infection	8.741 (1.936–39.476)	0.005*
*Aeromonas* spp. polymicrobial infection	0.610 (0.136–2.726)	0.517
Coinfection with anaerobic pathogens	0.518 (0.039–6.958)	0.620
Coinfection with *Clostridial* spp.	1.151 (0.061–21.699)	0.925
*Aeromonas* spp. resistant to cephalosporins	2.679 (0.635–11.296)	0.180
Initial ineffective empirical antimicrobial usage	5.798 (1.247–26.951)	0.025*
Systemic symptoms/signs		
Tachypnea	1.635 (0.374–7.155)	0.514
Shock	5.926 (1.254–28.006)	0.025*
Limbs symptoms/signs		
Hemorrhagic bullae	1.578 (0.414–6.011)	0.504
Skin necrosis	4.575 (1.190–17.597)	0.027*

Abbreviations: ^a^OR, odds ratio; ^b^CI, confidence interval.

## Discussion

Most *Aeromonas* SSTIs are associated with environmental exposure and are particularly related to traumatic occupational injuries or unexpected contact via recreational sporting activities^27,28^. This mode of acquisition results in soft tissue *Aeromonas* infections occurring more commonly during the summer season^25^. Our study was consistent with this finding.

*Aeromonas* SSTIs are often polymicrobial and frequently involve coinfections with other gram-negative rods or *Clostridium* species^25,27,30^. In studies of *Aeromonas* SSTIs or bacteremia, *A. hydrophila* was the most common pathogen isolated, and encompassed 47~69% *Aeromonas* infections^26,27,29^. In our results, *A. hydrophila* was also the most common infectious organism detected (67.6%), and *Clostridium* species were the most common coinfection pathogens.

According to past reports, *Aeromonas* and *Clostridial* necrotizing soft tissue infections were consistently associated with poor outcomes^7,19,31^. However, our patients exhibiting monomicrobial *Aeromonas* NF had a significantly higher mortality rate than those with polymicrobial *Aeromonas* NF. Another interesting phenomenon is that *Aeromonas* NF patients coinfected with *Clostridial* species also had better outcomes. On the other hand, we found *Aeromonas* NF combined with bloodstream infection (BSI) had significantly increased the mortality rate. Monomicrobial *Aeromonas* NF with bacteremia has commonly been reported to be associated with liver cirrhosis and malignancy that can rapidly impair the phagocytic activity of the reticuloendothelial system and result in septic shock and multiple organ failure^14,27,32^. Thus, we should pay more attention and aggressive treat those NF patients with *Aeromonas* bacteremia and monomicrobial infection.

Most *Aeromonas* strains are resistant to ampicillin, amoxicillin, and amoxicillin–clavulanate, whereas most are susceptible to sulfa drugs, fluoroquinolones, second- to fourth-generation cephalosporins, aminoglycosides, carbapenems, and tetracyclines^26,28,33,34^. Therefore, *Aeromonas* SSTIs may best be treated empirically with fluoroquinolones and/or a third- or fourth-generation cephalosporin or a carbapenem; however, a higher cephalosporins-resistance rate was found in the non-survival group (Table 3). Culture-directed antimicrobial therapy should be aggressively administered in such *Aeromonas* NF patients to avoid delayed use of appropriate antimicrobial therapy^11,13^.

A significantly higher mortality rate was observed in NF patients that also exhibited CKD, hepatic dysfunction, diabetes mellitus, and cancer^3,6,7,10,19,20,26^. Our *Aeromonas* NF patients with CKD or chronic hepatic dysfunction were present in statistically significantly higher numbers in the non-survival compared with the survival group. The non-survival group exhibited high severity of disease and 95% of patients required admission to the ICU. A delay in the first surgical intervention from symptom onset to the operating room of >24 h, as well as having advanced age, adversely affected survival outcome^3,20,26^. A delay in surgery of more than 24 h accounted for 30% of the patients within the non-survival group, and the mean age was >60 years old within these two groups.

**Figure 2:**
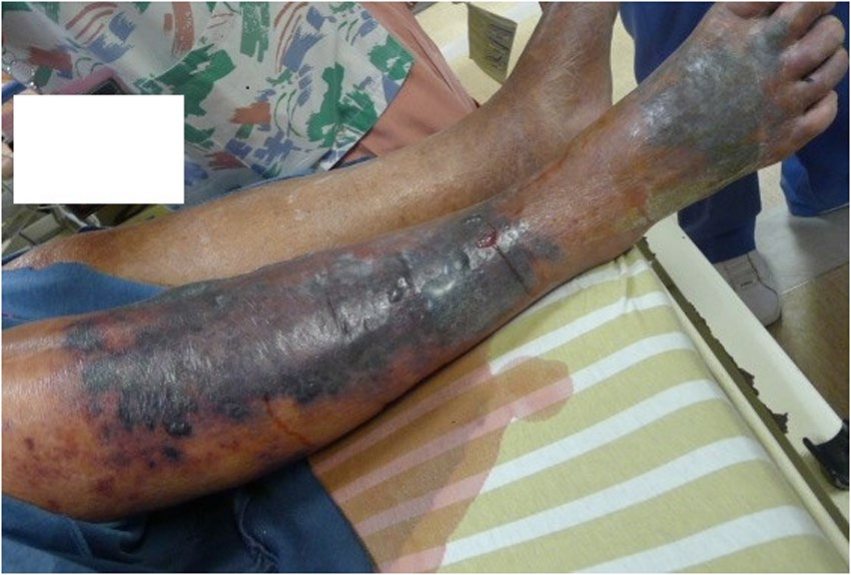
A 82 year-old male with a history of hepatitis B and old stroke had right low leg and foot pain on second day after a contused injury of toes. The right lower leg revealed patchy purpura, vesicles and skin necrosi with serous fluid soaking on the bed in the emergency room. After fasciotomy, the culture of blood and wound specimens confirmed *Aeromonas hydrophila*, however, this patient died on the 3rd day after admission owing to progressive septic shock and multiple organ failure.

In our study, the non-survival group contained a greater proportion of patients with hemorrhagic bullous lesions (60% vs. 29.2%) and skin necrosis (55% vs. 16.7%) than the surviving group, but multivariate logistic regression analysis revealed that non-survival patients presented associated with skin necrosis (*p* = 0.027). As the ischemic necrosis of the skin evolves, gangrene of the subcutaneous fat, dermis, and epidermis, manifesting progressively as bullae formation, ulceration and skin necrosis^14,35^. Hemorrhagic bullae and skin necrosis were also the late stage signals of necrotizing fasciitis^3,35,36^. Then, hemorrhagic bullae with skin necrosis appearance may increase the incidence of mortality (Fig. 2). Nonetheless, the emergence of hemorrhagic bullae would be considered a feature and an early independent predictor of mortality of *Aeromonas* NF.

In this study, the non-survival group exhibits a statistical tendency to have tachypnea and initially present with septic shock more than those within the survival group in univariate logistic analysis; however, multivariate logistic regression analysis bacteremia and shock revealed significant differences. Some literatures reported that initial presentations of tachypnea and hypotensive shock were also predictors for a poor outcome in NF patients^7,8,10,19^. This may explain to the fact that the death group presented more septicemia-related systemic inflammatory response symptoms to induce respiratory failure and shock.

Leucopenia, increased counts of banded leukocytes, thrombocytopenia, and severe hypoalbuminemia can be considered the clinical and laboratory risk indicators to initialize surgical intervention and to predict mortality for NF^3–8,15,20,21^. The non-survival group was associated with a higher rate of patients exhibiting leucopenia, leukocyte band cells >10%, lymphocytopenia, anemia, and thrombocytopenia compared with the survival group (Table 5). Lower serum albumin levels, prolonged PT, and higher total bilirubin levels were also noted within the non-survival group. These laboratory findings within *Aeromonas* NF infections are compatible with the aforementioned previous studies.

Multivariate logistic regression analysis revealed the initial ineffective empirical antimicrobial usage are related to poor outcomes for *Aeromonas* NF patients. Early prompt fasciotomy combined with appropriate antimicrobial therapy has been aggressively performed for patients highly suspected of having NF in our institution^8,13,14,17,19^. In our experience within the ICU, antimicrobial stewardship program and on-the-spot education by physicians specialized in infectious disease can potentially decrease sepsis-related and overall infection-related mortality rates by 54% and 41%, respectively^11^. The initial clinical presentation of *Aeromonas* NF is very similar to *Vibrio* NF, and especially within southern Taiwan, there may be a history of contact with dirty water or fish exposure. To treat these fulminate diseases, third- to fourth-generation cephalosporins combined with tetracycline or fluoroquinolones were commonly the empiric prescription before the infectious pathogens were identified^5,37^. In the non-survival group, we found 40% of *Aeromonas* species resistant to cephalosporins and 20% to tetracycline but only to 5% fluoroquinolones. So, we consider prescribing third- to fourth-generation cephalosporins combined with tetracycline and fluoroquinolones for highly suspected fulminate *Vibrio* or *Aeromonas* NF of limbs within the setting of our hospital. After the pathogens were identified, the antibiotic regimens were adjusted as necessary according to the patient’s clinical condition and results of the antibiotics drug sensitivity tests.

The present study was limited by having only 68 patients in a period of 18 years. However, to our knowledge, these are the largest patient numbers within such a study that can be currently found within PubMed. Another limitation contained within is that due to the long study period, some cases, medical records, and laboratory data had been lost and were not able to be recovered.

In conclusion, *Aeromonas* spp. NF of limbs is very rare and exhibits resistance to multiple antibiotics. NF caused by *Aeromonas* spp. revealed high mortality rates, even through aggressive surgical debridement and antibacterial therapies. Identifying those independent predictors, such as bacteremia, shock, progressive skin necrosis, monomicrobial infection, and application of the effective antimicrobial agents against *Aeromonas* under the supervision of infectious doctors, may improve clinical outcomes.

## Materials and Methods

### Study design and setting

This is a retrospective study performed by the VTR Group at CGMH-Chiayi from December 2001 to November 2019. We analyzed those patients admitted to the emergency department (ED) that were diagnosed with NF of limbs with undergoing surgical intervention, and a total 68 of patients were surgically and pathologically confirmed to have *Aeromonas* NF of limbs were included.

## Definitions

Patients with *Aeromonas* NF of limbs were enrolled in the study using the following criteria: (1) NF was defined either through histopathologic or surgical findings, such as the presence necrosis of the skin, subcutaneous fat, superficial fascia, or underlying muscles; (2) *Aeromonas* spp. was detected via isolation from soft tissue lesions and/or blood collected immediately after a patient’s arrival at the ED or during surgery; and (3) these bacteria infected any limb^3,6,17,22^.

Monomicrobial infection was documented by isolating single pathogenic bacteria as described above in criteria (2)^6,17^. Polymicrobial infections were documented in patients from whom *Aeromonas* isolates in addition to other bacterial pathogens were isolated from soft tissue lesions and/or blood samples. Ineffective empirical antimicrobial usage was defined as the administration of antimicrobial regimens that may be ineffective against *Aeromonas* isolates according to antimicrobial susceptibility testing when patients arrived ED^11,23^.

### Microbiology laboratory procedures

Gram-negative isolates that tested positive for cytochrome oxidase, glucose fermentation, citrate usage, indole production, and ornithine decarboxylase were classified as *Aeromonas* species. All strains were identified to the species level by conventional methods and were further verified by the API-20E and ID 32 GN Systems (bioMérieux Inc., Hazelwood, MO, USA), or the Vitek 2 ID-GNB identification card (bioMérieux Inc., Durham, NC, USA). These antimicrobial susceptibility tests were performed as recommended by the Clinical and Laboratory Standards Institute (CLSI), and the results were interpreted according to the CLSI criteria for these microorganisms.

### Demographic data, clinical presentations, and laboratory findings

Patients with *Aeromonas* NF of limbs were categorized within survival and non-survival groups. Data such as demographics, comorbidities, presenting signs and symptoms, laboratory findings, microbiologic results, initial antibiotics usage, and treatment outcomes were recorded and compared.

### Accordance

All procedures performed in studies involving human participants were in accordance with the ethical standards of the institutional and national research committee and with the 1964 Helsinki declaration and its later amendments or comparable ethical standards.

### Informed consent

All participants provided their written informed consent following the protocols approved by the Institutional Review Board of Chang Gung Medical Foundation. In accordance to the ethical approval, consents were not required from deceased patients’ relatives.

### Statistical analysis

The predictors for mortality were determined using a multivariate logistic regression model. Categorical variables were tested by Fisher’s exact test, continuous variables were tested by Student’s *t*-test or the Mann–Whitney U test, and a two-tailed *p*-value of <0.05 was considered statistically significant. Odds ratios (ORs) and 95% confidence intervals (CIs) were calculated to evaluate the strength of any association, as well as the precision of the estimated effect. All statistical calculations were performed using the Statistical Package for the Social Sciences for Windows, version 18.0 (Chicago, IL, USA).

### Ethics approval and consent to participate

This study protocol was approved by the Institutional Review Board of Chang Gung Medical Foundation (Number: 201801530B1B0).

## Data Availability

All datasets are available from the first author on reasonable request.
